# Socio-structural determinants of burn injuries in Africa: The role of social inequality, informal housing, and access to clean cooking energy technology

**DOI:** 10.1371/journal.pone.0336633

**Published:** 2025-12-12

**Authors:** Paul Terhemba Iorember, Ashley Van Niekerk

**Affiliations:** 1 Institute for Social and Health Sciences, University of South Africa, Johannesburg, South Africa; 2 Violence, Injury and Social Asymmetries Research Unit, South African Medical Research Council and University of South Africa, Cape Town, South Africa; Dankook University, KOREA, REPUBLIC OF

## Abstract

In many African countries, burn injuries often lead to chronic disability, psychological trauma and socio-economic hardship. These consequences have a long-term impact on people’s well-being and livelihoods. It is argued that the contributions of selected socio-structural determinants to burns are an important focus especially for population prevention measures. Therefore, this study examines the contribution of key socio-structural determinants to the non-fatal burden of burn injuries in Africa, with a focus on social inequality, informal housing and access to clean cooking energy technologies. The study applies the panel correlated standard errors regression, ordinary least squares regression with robust standard errors with adjusted predictions and marginal effects plots, and the least squares dummy variables analysis using panel data of all five regions of Africa for the period 2000–2022. The study finds that social inequality and informal housing exacerbate the burden of burn injuries in Africa. Conversely, access to clean cooking energy technology reduces this burden. The findings are confirmed by robustness checks. In this regard, the study recommends policies that lower structural inequities to reduce the risk of burns for vulnerable populations. This involves concretizing strategies to reduce poverty and providing more appropriately targeted social safety nets. There is also a need for region-specific strategies that tackle inequality, improve energy accessibility, and enhance housing conditions as a means of mitigating burn-related hazards across Africa.

## 1. Introduction

The world over, about 180,000 burn injury deaths annually occur, predominantly from low-middle income regions such as Southeast Asia and Africa (World Health Organisation [1] However, mortality grossly underrepresents the full burden, with an estimated 2.9 million inpatient injuries, and 31 million burn outpatients [2], with women, children, and the urban poor especially suffering non-fatal but debilitating injuries that are considered preventable [3]. More so, patients recovering from burn injuries frequently experience systemic inflammation and compromised immune function, which pose significant challenges for immediate and longer-term clinical management, nursing care and recovery [4].

The occurrence of burn injuries in Africa is especially common in informal settlements or urban slums which have a high population density and dependence on unclean and unsafe fuels for domestic use [5]. The inaccessibility of clean energy cooking technologies by the residents in these areas exposes them to high risks of fires and explosions. This exposure is worsened by the limited prevention programming and inaccessible or limited acute care and rehabilitation services [6]. The International Energy Agency (IEA) [7] reports that about 600 million people in Sub-Saharan Africa have limited access to clean energy such as electricity, and the majority of the population (about 80%) is still heavily dependent on traditional biomass or paraffin for cooking, heating and lighting, despite the documented contribution of such fuels to household fires and severe burn injuries, but also other deleterious health outcomes [8,9].

Recent evidence suggests that factors such as income inequality, poverty and inadequate or informal housing have an influence on burn injuries, particularly in low- and middle-income countries [1]. High levels of income inequality and widespread poverty create overcrowded and energy impoverished domestic environments where burn risks are amplified for vulnerable populations [10,11]. Income disparities and high rates of poverty furthermore influence access to clean cooking technologies as well as the type of housing. As a result, poor households often rely on the cheaper but unsafe fuels such as paraffin, charcoal, wood, and crop residues that are often used in makeshift or unstable appliances [5,8]. Furthermore, many houses in informal settlement areas are often made of flammable materials such as scrap wood or plastic sheeting, and lack proper electrical wiring, cross ventilation, and appropriate cooking places [12,13]. Therefore, sparks from makeshift wiring and electrical connections, faulty kerosene lamps, or from open flames from sources such as kerosene stoves and candles can trigger large fires with devastating effects. Poor ventilation also raises the risk of inhalation injuries and exacerbates post-burn complications and other health conditions. More so, there is limited access to fire and burn prevention and control equipment such as fire extinguishers or first aid kits in these communities and there is often limited knowledge of burn prevention or treatment, compounding the consequences of any fire or burn incident [14].

Given this milieu, this study examines key socio-structural determinants of burn injuries in Africa, focusing on the role of social inequality measured by the Palma ratio, informal housing, and access to clean cooking energy technologies. This integrated approach, rather than investigating the roles of such determinants separately or in isolation as in previous studies [10,8,15,16], provides a more comprehensive perspective to the impact of these socio-structural determinants on the burn injury burden. More so, this approach is important in the African context, where structural vulnerabilities may overlap and amplify one another, thereby demanding holistic, multi-dimensional solutions for sustainable interventions. The findings aim to inform sustainable development goals (SDG) aligned policy actions targeting health (SDG-3), clean energy (SDG-7), reduced inequalities (SDG-10), and sustainable urban development (SDG 11) in the African context.

This study departs from the extant literature which has extensively used mortality as the principal indicator of the burn injury burden [10,17], although this focus provides only the severest burden of burn injuries and neglects non-fatal outcomes. While mortality measures are important, it overlooks the substantial and often prolonged non-fatal burden and its consequences on wellbeing and livelihood, particularly in the African context. In many African countries, burn injuries more commonly result in chronic disability, psychological trauma, and socio-economic hardship rather than immediate death [1]. Therefore, using non-fatal indicators like incidence and prevalence presents a more obvious and policy-relevant picture of the overall burden of burn injuries. These indicators account for the frequency and persistence of burn-related health conditions and are argued to better inform prevention and intervention policies [3,1].

**Fig 1:**
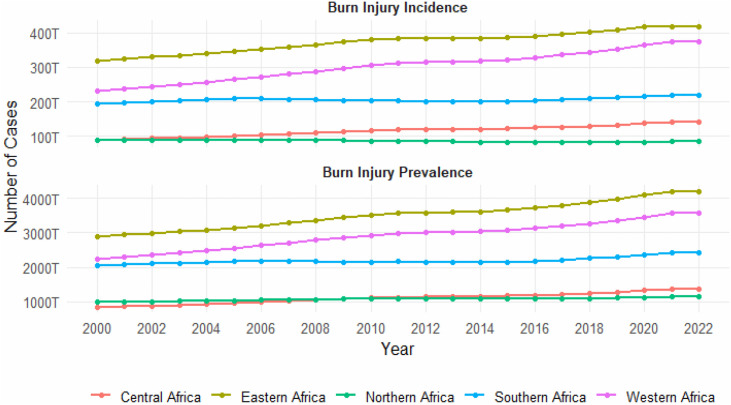
Spatio-temporal trends of burn injuries across Africa Regions. **Note: T = thousands**.

Fig 1 presents the spatio-temporal trends of the non-fatal burden of burn injuries (incidence and prevalence) across the African regions between 2000 and 2022. Evidenced from Fig 1 is that both incidence and prevalence show upward trajectories, with the highest burden observed in Eastern Africa, followed by Western Africa, with consistently high outcomes for all throughout. Southern Africa shows more moderate but increasing outcomes, while Central Africa and Northern Africa regions maintained comparatively lower outcomes over time. Furthermore, the prevalence of burn injuries is rising faster than the incidence rate, which suggests improvements in survival rates but also indicates weakness in prevention and rehabilitation. That is, while fewer people are dying from burn injuries, there are many more new cases, and thus many more survivors that increasingly experience long-term problems due to inadequate rehabilitation services. This changing public health scenario highlights this study’s importance, as it seeks to account for the role that socio-structural factors play in the continent’s changing burn burden. The study provides valuable insights for policymakers and healthcare professionals seeking to develop effective injury prevention programmes and comprehensive healthcare systems. It also provides evidence that supports region-specific and tailored interventions.

## 2. A review of studies on social inequality, informal housing, energy appliance risks and burns

The literature on social inequalities and burn injuries comprises mainly reviews with few empirical studies, with the latter primarily using descriptive and basic statistics. This study reviewed related studies focusing on social inequalities, fuel and energy technology risks, and informal housing in relation to burn injury, to clarify the current research gap.

Studies on social inequality and burn mortality have highlighted significant associations between low-income [18] or low socio-economic status [19,16,20,21] and burn injury. Some of this research has specifically focused on child burn mortality in low-income countries [17], with vulnerable subgroups including rural children with behavioral disorders [11]. Other studies, e.g., by Zoni et al. [22] and Heng et al. [23], have specifically identified spatial inequality and income deprivation as determinants of higher burn injury rates.

Studies on cooking energy, e.g., Kimemia et al. [24], have highlighted the limited effectiveness of even government certified kerosene (or paraffin) stoves, e.g., in South Africa, and reported that stove mishaps from both certified and other non-certified kerosene stoves are a major cause of severe burn injuries. Wanjeri et al. [25] examined the risk factors for burn injuries among patients hospitalized at Kenyatta National Hospital and also identified the use of kerosene for cooking and the lack of knowledge of burn injury prevention and fire safety as critical risk factors for burn injury. Other studies further afield, e.g., from South-East Asia, also highlighted stove-induced cooking burn injuries as comprising the majority of all burn injuries, with kerosene most commonly used [26]. The use of kerosene, with inadequately built and unstable appliances especially when used in low-income informal settlements, was identified as a leading contributor to burns [24] including childhood burns [8]. Thus, with the dangers of fossil fuel-based energies used heating, cooking and lighting, the need for safer, off-grid alternatives has increasingly been recognized [27,5]. In South Africa, Van Niekerk et al. [5] make a policy case linking energy poverty, domestic paraffin removal or control, and the lack of access to alternative and cleaner energies and technology with long-term trauma, and preventable mortality. However, while cleaner fuels like LPG and methanol are being promoted in a number of settings, Nyanana et al. [15] identify potential safety concerns. Kimemia and Van Niekerk [28] for example confirm the efficacy of methanol as an alternative to paraffin but also recognize that there is a serious risk of poisoning if methanol is not handled or packaged correctly. Wright et al. [29] make a case for more comprehensive strategies that involves technology, policy, and community engagement to ensure sustainable adoption of clean cooking fuels. Puzzolo et al. [30] identify the benefits of moving to clean cooking, including enhanced health conditions and economic upliftment, especially for women. Similarly, Bruce et al. [31] support a full transition to clean fuel given the gross inadequacy of even improved biomass stoves.

Studies on the role of informal housing to burns also indicate a very high incidence of burns in settings with a high concentration of such housing, particularly among children and women, as, e.g., indicated by Wong et al. [12] in a surveillance study in Kibera, Kenya. Their findings identify population density, crowded households and the use of unsafe cooking stoves and lamps as escalators of burn injuries. Govender et al. [9] and Kimemia & Van Niekerk [8] investigated domestic burns in South Africa and linked energy poverty and substandard housing to heightened burn incidence. Van Niekerk et al. [32] used an ecological analysis in Cape Town to significantly associate poor housing conditions, child dependency, and socio-economic barriers with childhood burns. Liao et al. [33], as part of an ongoing ethnographic study, synthesizes insights from participant observations to identify various housing-related issues such as smooking and hoarding as contributors to daily residential fire risks. Similarly, Tusiime et al. [13] report that the high prevalence of burns among children younger than five years is associated with congestion, the use of charcoal stoves and open cooking conditions in Kampala, Uganda.

### 2.1. Research gap

A review of empirical studies on the determinants of burn injuries reveals that while the extant literature has considered both fatal and non-fatal indicators of burn injuries, there is a notable emphasis on fatal indicators (i.e., mortality rates) as the primary measure of the burn injury burden. However, this focus represents only the severest burden of burn injuries unlike non-fatal indicators (i.e., incidence and prevalence) with such injuries more common and the related indicators better representing the prolonged consequences of burns on livelihood, particularly in the African context. This study therefore fills this research gap by using both the incidence and prevalence of burn injuries to account for the frequency and persistence of burn-related health conditions. Furthermore, most existing studies isolate the influence of income inequality or socio-economic status, without fully capturing the broader spectrum of social inequality. Specifically, there is limited empirical research that operationalizes social inequality using a multidimensional approach that captures key socio-economic indicators. This study fills this research gap by using the Palma Ratio, which measures social inequality by comparing the income share of the richest 10% of the population with that of the poorest 40%, highlighting disparities between the extremes of society. Methodologically, most studies on burn injuries are non-empirical, relying heavily on systematic reviews while a few others use descriptive or basic statistical methods, with limited use of advanced econometric modelling. As a result, causal inferences and the dynamic interplay between determinants such as institutionalized inequality, energy access, and socio-economic structures are often underexplored or weakly substantiated. The current study addresses this limitation by employing robust panel econometric techniques capable of accounting for heterogeneity, and cross-sectional dependence offering more reliable and policy-relevant findings for the African continent.

## 3. Material and methods

### 3.1. Data

This study uses a balanced panel dataset that consists of the five regions of Africa (Central, Eastern, Northern, Southern, and Western Africa) spanning the period from 2000 to 2022. The choice of this time period is based on data availability, as detailed and reliable data on the study variables are only available for this period. Most data started counting in 2000 and most are only available up to 2022. The University of South Africa’s College of Human Sciences Research Ethics Committee approved the study (reference number: 9267). S1 Appendix provides a detailed description of the variables, their measurement and the sources for variables used in the study. Following Usman et al. [34], we transform the variables by taking their natural logarithms to address the heteroscedasticity problem.

As the study focuses on African regions, the data has been sourced and aggregated so that each region (Central, Eastern, Northern, Southern and Western Africa) has a single value per year across the study period. This represents the average for all countries in each region over the study period. Aside from data on burn injuries, which is already reported on a regional basis by the Global Burden of Disease (GBD) study, the other variables are sourced on a country-by-country basis and aggregated using the formula:


$$Varname_{r,t}=\frac{\sum_{i=\text{1}}^{N_{r}}Varname_{i,t}}{N_{r}}\;\;\;\;\;\;\;\;\;\;\;\;\;\;\;\;\;\;\;\;\;\;\;\;\;\;\;\;\;\;\;\;\;\;\;\;\;\;\;\;\;\;\;\;\;\;\;\;\;\;\;\;\;\;\;\;\;\;\;\;\;\;\;\;\;\;\;\;\;\;\;$$ (1)

Where

Varname denotes name of a variable; r is region (e.g., Central Africa); i is country within region r; t is year and $N_{r}$ is number of countries in region r with data for year t.

Table 1 presents the descriptive statistics of the dataset by region and by panel. The panel dataset spans 23 years (2000–2022) across 5 regions (Central, Eastern, Northern, Southern and Western Africa regions) making for 115 total observations. The results show that Eastern Africa has the highest burn injuries incidence (BUIJinc) and burn injuries prevalence (BUIJpre), while Northern Africa has the lowest burden as indicated by the two indicators. In terms of social inequality (SOCI), Southern Africa has the highest values (i.e., the worst case) followed by Central Africa. The inequality in Northern Africa is relatively low compared to the other regions. Access to clean cooking energy technology (ACET) is highest in Southern Africa and lowest in Western Africa. The concentrations of informal housing (IHOS) is highest in Eastern Africa and this is closely followed by Central Africa and Western Africa, while Northern and Southern Africa regions have the lowest values. Regarding international trade (INET), only Southern Africa has a positive value indicating a net exporting position, while the other regions have negative values suggesting a net importing position.

**Table 1:** Descriptive Statistics.

Region	BUIJinc	BUIJpre	SOCI	ACET	IHOS	INET
	Mean	Mean	Mean	Mean	Mean	Mean
Central Africa	11.644	13.899	6.558	3.348	4.129	−2.405
Eastern Africa	12.828	15.064	5.506	2.807	4.140	−0.355
Northern Africa	11.350	13.882	4.033	3.392	3.610	−0.492
Southern Africa	12.234	14.598	7.419	3.880	3.674	0.093
Western Africa	12.613	14.874	4.975	2.193	4.057	−0.108
Panel						
Mean	12.134	14.463	5.698	3.124	3.922	−0.653
Std. Dev.	0.572	0.5039	1.287	0.667	0.283	1.035
Skewness	−0.152	−0.129	0.315	−0.207	−0.869	−1.888
Kurtosis	1.515	1.469	2.408	1.928	2.685	6.222
Jarque-Bera	11.003	11.555	3.584	6.333	14.955	118.010
Probability	0.004	0.0031	0.167	0.042	0.001	0.000
Observations	115	115	115	115	115	115

The lower part of Table 1 presents the descriptive statistics for the panel dataset. The mean values for the five regions are reported across the variables. The standard deviations are relatively low for most variables, ranging from IHOS (0.283) to SOCI (1.287), suggesting that the data are fairly stable and do not exhibit wide volatility. This eliminates concerns about extreme dispersion that could bias parameter estimates. With respect to normal distribution, the results of the skewness show that the majority of variables are approximately symmetric. Specifically, the skewness values of BUIJinc (–0.152), BUIJpre (–0.129), ACET (–0.207) and SOCI (0.315) are close to zero, reflecting balanced distribution around the mean. Although mild asymmetry exists in IHOS (–0.869) and INET (–1.888), the predominance of near-symmetric distributions across the dataset supports the robustness of subsequent econometric analysis.

### 3.2. Methods and estimation procedure

**Fig 2:**
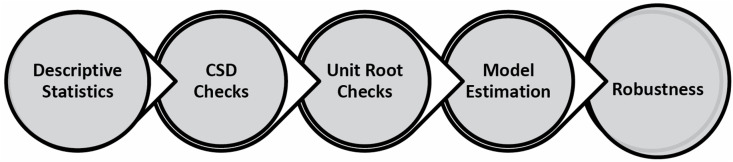
The estimation procedure.

The study adopts a five pronged methodological approach as shown in Fig 2. The figure outlines the five estimation procedures used in the analysis.

The first stage involved an examination of the correlation analysis of the study variables, using visual heatmaps to better understand the interrelationships among the variables used in the analysis. The second stage examines the cross – sectional dependence statuses of the variables using second and third generation weak CD test by Juodis and Reese [35], Pesaran and Xie [36, 37,38] and Fan et al [39]. All the four tests hypothesized a strong cross-sectional dependence in the panel. Rejecting the null hypotheses suggested the presence of cross-sectional dependence tests in the panel. Stage three involved checking the unit root properties of the variables using the [40] Cross-sectional Augmented IPS (CIPS) and Cross-sectional Augmented Dickey-Fuller (CADF). While the CADF test addressed cross-sectional dependence by augmenting individual ADF regressions with cross-sectional averages, the CIPS test extended the CADF by aggregating its statistics across units to produce a panel statistic that is equivalent to the average of the individual statistics. The fourth stage involved the main analysis using the Panel Corrected Standard Errors (PCSE) regression and Ordinary Least Squares (OLS) model with robust standard errors with adjusted predictions and marginal effects plots. In the fifth and final stage of the estimation, we conducted a robustness analysis using the Least Squares Dummy Variable (LSDV) fixed-effects model to account for unobserved heterogeneity across the regions and over time. The LSDV approach adds value to the study by isolating the regional variations and controlling for time-invariant characteristics. It also affirms the consistency of the main estimations which further implies that the observed relationships are not driven by omitted regional attributes or temporal shocks.

### 3.3. Model specification and estimation

To achieve the objectives of the study, two models were specified and estimated using various estimation techniques. The first model represented in equation (2) captures the effect of the structural determinants on the incidence of burn injuries and the second model represented by equation (3) reflects the effect of the structural determinants on the prevalence of burn injuries. The panel specifications of the models are as follows;


$$BUIJinc_{it}=\alpha +\beta_{\text{1}}SOCI_{it}+\beta_{\text{2}}ACET_{it}+\beta_{\text{3}}IHOS_{it}+\beta_{\text{4}}INET_{it}+\varepsilon_{it}\;\;\;\;\;\;\;\;\;$$ (2)

$$BUIJpre_{it}=\varphi +\vartheta_{\text{1}}SOCI_{it}+\vartheta_{\text{2}}ACET_{it}+\vartheta_{\text{3}}IHOS_{it}+\vartheta_{\text{4}}INET_{it}+\mu_{it}\;$$ (3)

Where the variables remain as defined in Table 1. α and φ are the intercept terms. $\beta_{\text{1}}-\beta_{\text{4}}$ and $\vartheta_{\text{1}}-\vartheta_{\text{4}}$ are the parameters for equations 2 and 3 respectively.

Three techniques of estimations were applied to investigate the relationships in equations (2) and (3). First, the study applied the Panel Corrected Standard Errors (PCSE) regression to estimate equations (2) and (3). The PCSE regression proposed by [41]  estimates linear cross-sectional models using OLS or Prais–Winsten regression and computes the standard errors and the variance–covariance estimates. It assumes that the disturbances are heteroskedastic and contemporaneously correlated across panels. It is particularly suitable for estimating panels with evidence of cross-sectional dependence and where the number of time periods (*T*) is higher than the number of cross-sectional units (*N*) as the case with the current study with T = 22 (2000–2022) and *N* = 5 (the five African regions).

Second, we estimated equations (2) and (3) using the Ordinary Least Squares (OLS) model with robust standard errors and report adjusted predictions and marginal effects, visualized with 95% confidence bands. Specifically, the adjusted predictions place effects directly on the outcome scale, improving interpretability and policy relevance while the marginal-effects plots show how the conditional slope varies across the focal regressor, revealing heterogeneity that cannot not be seen in single coefficients. We evaluated predictions over a grid within the observed support to avoid extrapolation, and used confidence bands to convey statistical uncertainty. This procedure aligns with the established guidance on adjusted predictions and marginal effects in Stata’s margins/marginsplot framework [42,43].

Third, we applied the Least Squares Dummy Variable (LSDV) fixed-effects model to check for the robustness of the findings and to analyse the heterogeneity across the African regions and over time. In this model, the dependent variables (BUIJinc and BUIJpre) represented by $Y_{it}$is modelled as a function of the independent variables (SOCI, ACET, IHOS, INET) captured by $X_{it}$, regional-specific effects, and year-specific effects is given as:


$$Y_{it}=\delta +\pi X_{it}+\sum_{i=\text{1}}^{N-\text{1}}\gamma_{i}D_{i}+\sum_{t=\text{1}}^{T-\text{1}}\emptyset_{t}{YR}_{t}+\epsilon_{it}\;\;\;\;\;\;\;\;\;\;\;\;$$ (4)

Where

$D_{i}$ is the regional dummies (region fixed effects) and ${YR}_{t}$ is the year dummies (year fixed effects). $\gamma_{i}$ is the time-invariant regional characteristics such as institutions and cultures, and $\emptyset_{t}$ is the shocks common to all regions in a given year such as pandemics. That is, $\gamma_{i}$ and $\emptyset_{t}$ are the parameter estimates for region and year effects respectively. δ and π are the intercepts and slope coefficients of the LSDV model respectively, and $\epsilon_{it}$ is the error term. The inclusion of $\gamma_{i}$ and $\emptyset_{t}$ ensures that the estimates of π are unbiased by unobserved heterogeneity that is either constant across time within a region or common across all countries within a year.

## 4. Results and findings

### 4.1. Preliminary analysis

**Fig 3:**
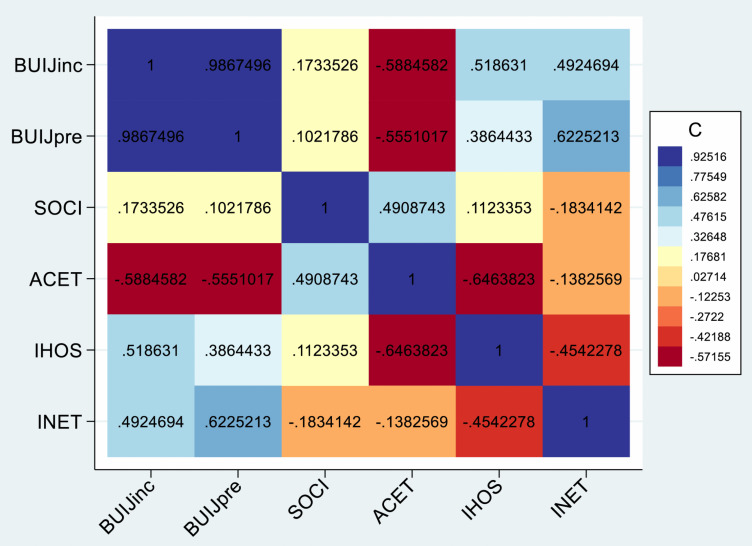
Heatmap visualizing pairwise correlation among the panel variables.

In the first stage of the analysis, we visualize the correlation matrix to better understand the interrelationships among the variables used in the analysis using correlation heatmaps with the results shown in Fig 3. The colour shading in Fig 3 reflects the strength and direction of correlations, with warmer colours (blue-like) indicating positive relationships and cooler (red-like) colours indicating negative correlations. The value in each cell is the Pearson correlation coefficient which ranges from −1–1. The results show a weak to moderate correlation between the independent variables and the dependent variables indicating the absence of multicollinearity (i.e., the explanatory variables are not highly correlated) – a condition that is suitable for further analysis. Moreover, the weak to moderate correlations also indicate that the individual effects of the explanatory variables on burn injuries may not be fully captured by a simple bivariate analysis. To properly uncover these dynamics, we further apply multivariate modeling with dynamic and robust capabilities to explore indirect pathways through which socio-structural factors influence burn injury outcomes.

#### 4.1.1. Tests for cross-sectional dependence.

Further, from the correlation analysis we examined the cross-sectional dependence of the panel variables to check if shocks or policy changes in one or more region had a spillover effect to the other countries. This test is particularly important to determine whether the examination of the effects as a panel rather than individual regions is appropriate and whether the first generation panel analysis can be applied. To achieve this, we applied the second and third generation tests to examine the cross-sectional dependence (CSD) in the variables and the results are shown in Table 2. Evidently, the results reveal significant evidence of CSD for the variables, including burn BUIJinc, BUIJpre, SOCI, ACET, IHOS. However, moderate CSD is observed for INET. This suggests that shocks or policy changes to one region will have spillover effect on the others substantiating the interconnectedness of public health and structural factors across African regions.

**Table 2:** Weak CSD Test using Second and Third Generation Approaches.

CSD Test	Pesaran [37,38]	Pesaran & Xie [36]	Juodis & Reese [35]	Fan et al [39]	Interpretation
	CD	CD^*^	CDw	CDw+	
BUIJinc	3.18 ***	0.18	0.96	35.09***	Significant CSD
	(0.001)	(0.854)	(0.339)	(0.000)	
BUIJpre	13.62***	0.18	−2.99***	40.10***	Significant CSD
	(0.000)	(0.857)	(0.003)	(0.000)	
SOCI	9.32 ***	1.13	−2.07**	27.40 ***	Significant CSD
	(0.000)	(0.260)	(0.038)	(0.000)	
ACET	14.14 ***	(0.260)	−2.94***	41.79 ***	Significant CSD
	(0.000)	(0.399)	(0.003)	(0.000)	
IHOS	12.46 ***	0.26	−2.49**	36.91 ***	Significant CSD
	(0.000)	(0.793)	(0.013)	(0.000)	
INET	1.22	2.37**	1.31	31.83 ***	Moderate CSD
	(0.224))	(0.018)	(0.189)	(0.000)	

Note: () denotes p-values (p). (p < 0.01; 0.05; 0.10) statistical significance at 1, 5 and 10 per cent respectively

#### 4.1.2. Tests for Unit Root.

Given the presence of CSD, the study applies the Cross-sectional Im, Pesaran, and Shin (CIPS) and Cross-sectional Augmented Dickey-Fuller (CADF). Both the CIPS and CADF tests account for CSD while examining unit roots in the panel series. Their results in Table 3 show that the series have unit roots in level form and are therefore non-stationary at levels form. However, after differencing, the series became stationary in at least one of the tests, implying the absence of a unit root at the difference form. These findings suggest that subsequent empirical analysis should employ second-generation estimators that are robust to cross-sectional dependence.

**Table 3:** Results of Panel Unit Root Tests with CSD.

Variables	CIPS test		CADF test	
	Level form	Difference form	Level form	Difference form
BUIJinc	−0.677	−3.544***	−2.067	−3.857***
BUIJpre	−0.522	−2.278*	−1.034	−2.390**
SOCI	−1.511	−4.767 ***	−2.043	−1.972
ACET	−0.237	−5.881 ***	−1.117	−3.985***
IHOS	−3.559	−5.805 ***	−1.117	−2.277 *
INET	−1.723	−5.775 ***	−1.587	−5.417 ***
Critical valuesat				
1%	−2.57	−2.57	−2.57	−2.57
5%	−2.33	−2.33	−2.33	−2.33
10%	−2.21	−2.21	−2.21	−2.21

Note: The asterisks ***, ** and * imply statistical significance at the 1, 5 and 10 per cent levels, respectively.

### 4.2. Main analysis

The study employs the Panel Correlated Standard Errors (PCSE) and the Ordinary Least Squares (OLS) model with robust standard errors techniques to empirically investigate the selected socio-structural determinants of burn injuries in Africa. To ensure robustness, two models are estimated, one for the incidence of burn injuries and another for prevalence and the results presented in Table 4. The first two columns of Table 4 (1) present the results for burn injuries incidence while columns 3 and 4 of Table 4 (2) capture the results for burn injuries prevalence.

**Table 4:** Results of the Socio-Structural Determinants of Incidence of Burn Injuries in Africa using Panel Correlated Standard Errors (PCSE) Estimator. Dependent variable: Burn Injuries.

Dep/Indep.Variables	1		2	
	Incidence (BUIJinc)		Prevalence (BUIJpre)	
	Coeff.	Std.Err.	Coeff.	Std.Err.
SOCI	0.0794***	0.0236	0.0552***	0.0207
ACET	−0.1274	0.9003	−0.1207	0.0819
IHOS	0.9874***	0.2683	0.6544***	0.2501
INET	0.3697***	0.0513	0.3565***	0.0488
Constant	8.4486***	1.2206	12.1919***	1.1282
Wald	86.83***	0.000	109.54***	0.000
p-value	0.0000		0.0000	
R-squared	0.5920		0.5857	

Note: *** p < 0.01, ** p < 0.05, * p < 0.1; Dep = Dependent; Indep = Independent

Evidently, the PCSE results in Table 4 provide interesting and policy aligned findings. The results show that SOCI has a positive and statistically significant effect on both burn injuries incidence and burn injuries prevalence with coefficients and probability values of 0.0794 (p < 0.01) and 0.0552 (p < 0.01) respectively. This suggests that an increase in social inequality worsens burn injuries by increasing the incidence and prevalence cases. ACET shows a consistently negative but non-significant effect on both burn injuries incidence and prevalence, with coefficients of −0.1274 (p > 0.01) and −0.1207 (p > 0.01) respectively. While the negative effect confirms that improved access to clean cooking energy technology reduces the incidence and prevalence of burn injuries, it is not statistically significant. The evidence is therefore suggestive rather than conclusive, and should be taken with appropriate caution. IHOS exerts a positive and statistically significant effect on burn injuries incidence and prevalence with coefficients of 0.9874 (p > 0.01) and 0.6544 (p > 0.01) respectively, indicating that inadequate housing conditions due especially to overcrowding, unsafe building materials and unsafe energy use significantly contributes to burn injury risks. INET as a control variable has a significant positive effect on burn injuries incidence and prevalence with coefficients of 0.3697 (p < 0.01) and 0.3565 (p < 0.01) respectively. This suggests that increased energy trade is associated with higher burn injuries, possibly due to uneven domestic energy access and risk exposure.

**Fig 4:**
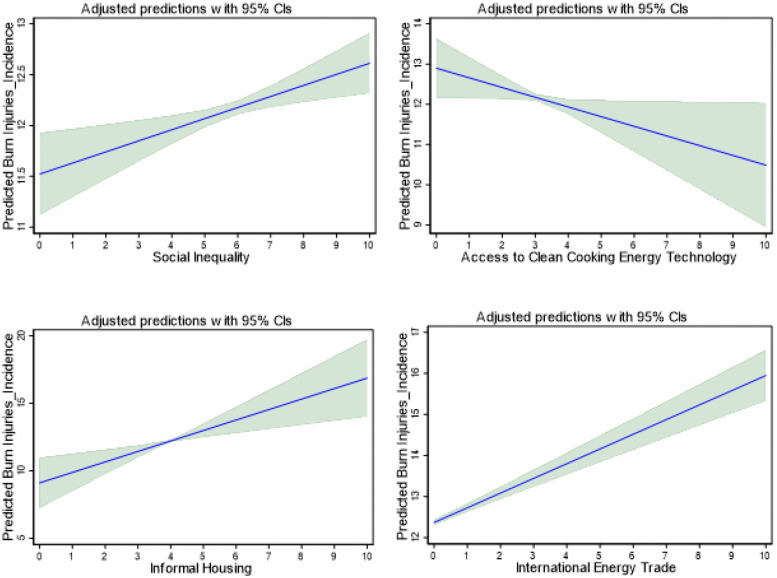
Marginal Effect Plots of the Effect of the Socio-Structural Factors on the Incidence of Burns Injuries.

**Fig 5:**
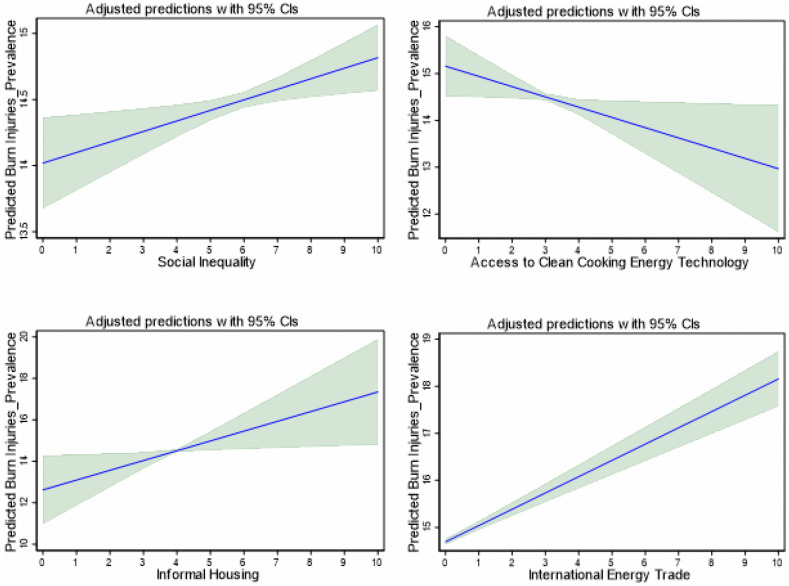
Marginal Effect Plots of the Effect of the Socio-Structural Factors on the Prevalence of Burns injuries.

Further, we apply the OLS with robust standard errors and report adjusted predictions and marginal effects, visualized with 95% confidence bands to confirm the marginal effects of SOCI, ACET, IHOS and INET on the incidence and prevalence of burn injuries. These effects are visualized in Fig 4 and Fig 5 respectively. The marginal effect plots further support the regression results. The plots of the marginal effects display significant evidence of increasing trend in burn injuries (incidence and prevalence) with increases in SOCI, IHOS and INET. These results align with the findings of the PCSE regressions in Table 4. More so, the marginal effects plots disclose a declining trend in burn injuries with an increase in ACET, as established by the findings in Table 4. Taken together, the findings stress the importance of social inequality, access to clean cooking energy technologies and housing conditions in shaping burn injury outcomes, while leveraging energy trade, to effectively reduce burn-related injuries in Africa.

### 4.3. Robustness Checks (using LSDV Regression with Region and Year Fixed effects)

To validate the robustness of the findings, we estimate the LSDV regression with region and year fixed effects with the results presented in Table 5. The findings are consistent with the PCSE results in Table 4 and the marginal effects plots in Fig 4 and Fig 5. SOCI, IHOS and INET exert increasing effects on burn injuries in Africa. Similarly, ACET shows a reducing effect on burn injuries consistent with the PCSE results in Table 4. While the effects of ACET and IHOS are significant for both burn injury incidence and prevalence, the effects of SOCI and INET are significant for the prevalence and incidence models, respectively.

**Table 5:** Results of the Robustness Checks using Least Square Dummy Variable (LSDV) Panel Regression.

Dep/Indep.Variables	Incidence (BUIJinc)		Prevalence (BUIJpre)	
	1	2	3	4
	Coeff.(Std. Err.)	Coeff.(Std. Err.)	Coeff.(Std. Err.)	Coeff.(Std. Err.)
SOCI	0.0188		0.4012***	
	(0.0153)		(0.0126)	
ACET	−0.0686**		−0.0500*	
	(0.0329)		(0.0272)	
IHOS	0.4035***		0.2239***	
	(0.0814)		(0.0673)	
INET	0.0172***		0.0117	
	(0.0103)		(0.0085)	
Eastern Africa		1.1269***		1.1532***
		(0.0377)		(0.3114)
Northern Africa		−0.0670		0.1797***
		(0.0638)		(0.0527)
Southern Africa		0.7501***		0.7633***
		(0.0454)		(0.0375)
Western Africa		0.9089***		0.9694***
		(0.6040)		(0.0499)
Regional Fixed Effects		Yes		Yes
Year Fixed Effects	Yes		Yes	
Observations	115	115	115	115

Note: *** p < 0.01, ** p < 0.05, * p < 0.1; Dep = Dependent; Indep = Independent

Regarding the region-specific effects, where Central Africa is omitted as the reference region. The LSDV treats the first cross section or region (d1) as the baseline. More so, given that the Central Africa region’s burden of burn injuries is not extremely high or low, we retain it as the baseline. The results in columns 2 and 4 of Table 5 indicate substantial heterogeneity. The Eastern, Southern and Western African regions have positive and significant coefficients of 1.1269, 0.7501 and 0.9089, respectively, in Model 2 of Table 5, indicating a persistently higher incidence of burn injuries than the baseline. Similarly, the higher coefficients of 1.1532, 0.7633 and 0.9694 in model 4 imply a persistently higher prevalence of burn injuries in the Eastern, Southern and Western African regions than in the baseline. However, the effect of Northern Africa is small in both cases compared to the baseline. For the year effects, the results show a steady and significant upward trend from 2003 onwards (the year fixed effects results can be provided on request). These findings show that both the region-specific structural factors and the general changes over time are important in explaining how the burden of burn injuries has evolved.

## 5. Discussion

The finding of a positive and statistically significant effect of social inequality on both the incidence and prevalence of burn injuries as evidenced by the PCSE and marginal effect analysis confirms that social inequality is a significant socio-structural factor in burn injuries in Africa. This finding aligns with the position of existing literature such as Dissanaike et al. [19], Sengoelge et al. [10] and Edelman [18], who consistently identified associations between different individual socio-economic indicators including income inequality and poverty rates with burn injuries. The socioeconomic contributors to such injuries have been explained by the ‘fundamental causes’ theory, which indicates that social and daily living resources are disproportionately distributed, with those of lower socio-economic status having fewer resources for health and wellbeing, specifically as regards finances, knowledge, social power and social networks [44] and thus less capacity and fewer opportunities to promote their safety and wellbeing [45]. The theory of ‘fundamental causes’ indicates that the association between socioeconomic status and health may differ across countries depending on the interventions, e.g., poverty alleviation and accessible health care policies, that a country may implement to reduce socioeconomic inequalities [44].

The positive and statistically significant coefficient of informal housing for both incidence and prevalence models indicates that inadequate housing is a strong determinant of burn injuries. Living in an informal and unplanned settlement has previously been reported as a significant risk factor to burn injuries [46,47]. Studies, e.g., by Tusiime et al. [13] and Wong et al. [12] highlighted the increased risk of young child burn injuries in informal settlements. These places typically comprise shack dwellings that lack approved electrical systems, safe cooking areas and fire-resistant materials. This creates a physical environment that is likely to result in fire accidents. The current finding echoes the views of Walls et al. [14] and Van Niekerk et al. [32], who associated the structural design, cramped spatial layout and dwelling materials used in informal housing with elevated fire hazards and an increased risk of burn injuries.

The current findings substantiate the importance of access to clean and safe cooking energy technology, with more limited access significantly associated with burn injuries. This finding confirms the protective influence of safe cooking energy in dampening the risks for burn injuries. More so, the lack of access to clean energy pushes households towards utilizing hazardous cooking and heating techniques like kerosene stoves and open flames, thereby increasing the risk of burns. This finding is consistent with reports by Govender et al. [9] and Kimemia and Van Niekerk [8] that energy poverty which is characterized by unsafe fuels and unstable appliances is a major public health concern, especially in informal settlements. Therefore, enhancing access to clean and safe cooking fuels is a critical burn injury prevention strategy. The finding also echoes those of studies from outside Africa such as by Mehta et al. [26], who reported the use specifically of kerosene stoves for cooking and the presence of open flames as major drivers of domestic burn injuries.

Notably, this study used energy trade as the control variable, which showed a significant positive coefficient on both incidence and prevalence of burn injuries. This finding suggests that simply increasing trade in energy resources, which has included significant fossil fuels in Africa, would thus not automatically enhance safe energy access and protect households from fires. It echoes the resource curse postulation that energy wealth and weak social and health outcomes can still co-exist, especially if governance and energy redistributive policies are not strong and geared towards protecting vulnerable groups (Bellinger & Fails, 2020; Van Niekerk et al., 2022b).

## 6. Conclusions and policy recommendations

This study examines key socio-structural determinants of burn injuries in Africa. It focuses on the collective impact of social inequalities, informal housing and access to clean cooking energy technologies, while controlling for the effects of international trade. The study utilizes robust panel data analysis having established the existence of cross-sectional dependence in the series. Specifically, the study applied the panel correlated standard errors regression, OLS model with robust standard errors with adjusted predictions and marginal effects plots, and the least squares dummy variables regression to account for unobserved heterogeneity across the regions and over time. Using the incidence and prevalence of burn injuries as non-fatal indicators of burn injuries, the findings show that social inequality and informal housing exacerbate the burden of burn injuries across all regions in Africa, signifying their criticality in efforts to mitigate the incidence of burn injuries on the continent. Conversely, access to clean cooking energy technology reduces the burden of burn injuries. More so, the international energy trade which serves as the control variable shows an increasing effect on the burden of burn injuries. These findings are corroborated by the results of the least squares dummy variables approach. Moreover, the robustness of these results reveal substantial heterogeneity across the regions with Eastern, Southern and Western African regions showing a persistently higher burden of burn injuries (BUIJinc and BUIJpre) than Central Africa while the effect of Northern Africa is, however, small relative to Central Africa.

Based on the findings, it is recommended that reducing burns in Africa requires strategies to promote the access to clean energy for all, reduce inequality and its most profound impacts of health and wellbeing, and strengthen access to safer housing for poor communities. Furthermore, there is a significant variation in burn injury outcomes across different African regions. Using Central Africa as a baseline, Eastern, Southern, and Western Africa consistently exhibit higher burn rates, indicating the need for region-specific treatments. In Eastern Africa, where burns effects are highest and social inequality and informal housing are serious issues, governments should prioritise the promotion of equity in educational, employment and health opportunities and improving access to safe housing. Southern Africa, with the highest level of social inequality and a high effect on burn injuries, requires redistributive reforms, strengthening of social and health safety nets, and improved housing safety regulations. Western Africa, with a high burden of burns and the lowest access to clean energy, requires an acceleration of its energy transition through regional financing and community-based programmes. In contrast, North Africa experiences fewer burn injuries, so policy initiatives should focus on maintaining fair energy access and fostering inclusive urban growth. Together, these findings emphasise the need for region-specific strategies that tackle inequality, improve energy accessibility, and enhance housing conditions as a means of mitigating burn-related hazards across Africa.

While the study makes significant contributions, it is constrained by the use of only non-fatal burden of burn injuries (incidence and prevalence) without accounting for the fatal burden (i.e., the mortality rate). However, this choice is appropriate in the African context where non-fatal burns are widespread and their consequences on wellbeing and livelihood often more consequential and prolonged. More so, the study does not address the varying capacities of regional or individual country health systems for burn treatment and long-term rehabilitation. Future research could examine how access to healthcare (including surgical care, psychosocial support and community reintegration) interacts with other structural determinants of burn injuries in Africa. This could be carried out on a country-by-country basis to see how health systems and socio-structural determinants influence health outcomes. Additionally, our study applies mainly mean based (PCSE, OLS with robustness and LSDV) methods of data analysis, which essentially address linear relationships. Further studies could use quantile and threshold regressions to capture nonlinearities and threshold effects that may exist in the model across different developmental levels.

## Supporting information

S1 Appendix Variables Description, Measurement and Sources. (DOCX)

## References

[1] World Health Organization (WHO). (2023). Burns [Fact Sheet]. Geneva: WHO. https://www.who.int/news-room/fact-sheets/detail/burns

[2] Haagsma JA, Graetz N, Bolliger I, Naghavi M, Higashi H, Mullany EC, et al. The global burden of injury: incidence, mortality, disability-adjusted life years and time trends from the Global Burden of Disease study 2013. Inj Prev. 2016;22(1):3–18. doi: 10.1136/injuryprev-2015-041616 26635210 PMC4752630

[3] Yin B, He Y, Zhang Z, Cheng X, Bao W, Li S, et al. Global burden of burns and its association with socio-economic development status, 1990–2019. Burns. 2024;50(2):321–74. 38102041 10.1016/j.burns.2023.02.007

[4] Barrett LW, Fear VS, Waithman JC, Wood FM, Fear MW. Understanding acute burn injury as a chronic disease. Burns Trauma. 2019;7(1):s41038-019-0163-2. 10.1186/s41038-019-0163-2 PMC6745803 31534977

[5] Van Niekerk A, Kimemia D, Seedat M, Annegarn H. Energy impoverishment and burns: The case for an expedited, safe and inclusive energy transition in South Africa. S Afr J Sci. 2022b;118(3/4). doi: 10.17159/sajs.2022/13148

[6] Smolle C, Cambiaso-Daniel J, Forbes AA, Wurzer P, Hundeshagen G, Branski LK, et al. Recent trends in burn epidemiology worldwide: A systematic review. Burns. 2017;43(2):249–57. doi: 10.1016/j.burns.2016.08.013 27600982 PMC5616188

[7] International Energy Agency IEA. Access to electricity. https://www.iea.org/reports/sdg7-data-and-projections/access-to-electricity. 2023.

[8] Kimemia DK, Van Niekerk A. Energy poverty, shack fires and childhood burns. S Afr Med J. 2017;107(4):289. doi: 10.7196/samj.2017.v107i4.12436

[9] Govender R, Kimemia D, Hornsby N, Van Niekerk A. Differentiation of paediatric burn injury by household energy source in South Africa. J energy South Afr. 2020;31(2):48–58. doi: 10.17159/2413-3051/2020/v31i1a8096

[10] Sengoelge M, El-Khatib Z, Laflamme L. The global burden of child burn injuries in light of country level economic development and income inequality. Prev Med Rep. 2017;6:115–20. doi: 10.1016/j.pmedr.2017.02.024 28316905 PMC5345966

[11] Padalko A, Cristall N, Gawaziuk JP, Logsetty S. Social Complexity and Risk for Pediatric Burn Injury: A Systematic Review. J Burn Care Res. 2019;40(4):478–99. doi: 10.1093/jbcr/irz059 30918946

[12] Wong JM, Nyachieo DO, Benzekri NA, Cosmas L, Ondari D, Yekta S, et al. Sustained high incidence of injuries from burns in a densely populated urban slum in Kenya: an emerging public health priority. Burns. 2014;40(6):1194–200. 24461306 10.1016/j.burns.2013.12.010 PMC4665976

[13] Tusiime M, Musoke D, Muneza F, Mutto M, Kobusingye O. Prevalence, risk factors and perceptions of caregivers on burns among children under 5 years in Kisenyi slum, Kampala, Uganda. Injury Epidemiology. 2022;9(1):18. 35689273 10.1186/s40621-022-00382-w PMC9188101

[14] Walls RS, Eksteen R, Kahanji C, Cicione A. Appraisal of fire safety interventions and strategies for informal settlements in South Africa. DPM. 2019;28(3):343–58. doi: 10.1108/dpm-10-2018-0350

[15] Nyanana A, Rwanyuma L, Chiwanga F, Mbwambo J, Pallangyo C, Tarimo U, et al. Cooking-related burn injuries at Muhimbili National hospital and knowledge about safe use of liquefied petroleum gas in Dar Es Salaam, Tanzania: A cross-sectional study. Burns Open. 2024;8(3):211–6. doi: 10.1016/j.burnso.2024.05.002

[16] Jacobs C, Vacek J, Many B, Bouchard M, Abdullah F. An Analysis of Factors Associated with Burn Injury Outcomes in Low- and Middle-Income Countries. J Surg Res. 2021;257:442–8. doi: 10.1016/j.jss.2020.08.019 32892143

[17] Wang K, Jiang C, Wu Q, Li Z. Trends and cross-country inequalities in global burns burden among children and adolescents: A population-based study from 1990 to 2021. Burns. 2025;51(3):107377. doi: 10.1016/j.burns.2025.107377 39848116

[18] Edelman LS. Social and economic factors associated with the risk of burn injury. Burns. 2007;33(8):958–65. 17869003 10.1016/j.burns.2007.05.002

[19] Dissanaike S, Ha D, Mitchell D, Larumbe E. Socioeconomic status, gender, and burn injury: A retrospective review. Am J Surg. 2017;214(4):677–81. doi: 10.1016/j.amjsurg.2017.06.012 28693838

[20] Mason S, Gause E, McMullen K, Murphy SC, Sibbett S, Holavanahalli R, et al. Impact of community-level socioeconomic disparities on quality of life after burn injury: a Burn Model Systems Database study. Burns. 2023;49(4):861–9. 35786500 10.1016/j.burns.2022.06.004 PMC10052954

[21] Snelling S, Challoner T, Lewis D. Burns and socioeconomic deprivation: the experience of an adult burns centre. Burns. 2021;47(8):1890–5. 33722449 10.1016/j.burns.2021.02.019

[22] Zoni AC, Domínguez-Berjón MF, Esteban-Vasallo MD, Velázquez-Buendía LM, Blaya-Nováková V, Regidor E. Socioeconomic inequalities in injuries treated in primary care in Madrid, Spain. J Public Health (Oxf). 2017;39(1):45–51. doi: 10.1093/pubmed/fdw005 26869695

[23] Heng JS, Atkins J, Clancy O, Takata M, Dunn KW, Jones I, et al. Geographical analysis of socioeconomic factors in risk of domestic burn injury in London 2007-2013. Burns. 2015;41(3):437–45. doi: 10.1016/j.burns.2014.12.001 25554260

[24] Kimemia D, van Niekerk A, Govender R, Seedat M. Burns and fires in South Africa’s informal settlements: have approved kerosene stoves improved safety?. Burns. 2018;44(4):969–79. 29395395 10.1016/j.burns.2017.11.006

[25] Wanjeri JK, Kinoti M, Olewe TH. Risk factors for burn injuries and fire safety awareness among patients hospitalized at a public hospital in Nairobi, Kenya: A case control study. Burns. 2018;44(4):962–8. 29395410 10.1016/j.burns.2017.11.007

[26] Mehta K, Thrikutam N, Hoyte-Williams PE, Falk H, Nakarmi K, Stewart B. Epidemiology and outcomes of cooking-and cookstove-related burn injuries: a World Health Organization global burn registry report. Journal of Burn Care & Research. 2023;44(3):508–16. 34850021 10.1093/jbcr/irab166 PMC10413420

[27] Mills E. Identifying and reducing the health and safety impacts of fuel-based lighting. Energy for Sustainable Development. 2016;30:39–50. doi: 10.1016/j.esd.2015.11.002

[28] Kimemia D, Van Niekerk A. Is methanol a clean, efficient, healthy and safe cooking solution for Africa? Experiences of benefits, challenges and prospects for diffusion. Energy for Sustainable Development. 2024;81:101498. doi: 10.1016/j.esd.2024.101498

[29] Wright C, Sathre R, Buluswar S. The global challenge of clean cooking systems. Food Sec. 2020;12(6):1219–40. doi: 10.1007/s12571-020-01061-8

[30] Puzzolo E, Pope D, Stanistreet D, Rehfuess EA, Bruce NG. Clean fuels for resource-poor settings: A systematic review of barriers and enablers to adoption and sustained use. Environ Res. 2016;146:218–34. doi: 10.1016/j.envres.2016.01.002 26775003

[31] Bruce N, Pope D, Rehfuess E, Balakrishnan K, Adair-Rohani H, Dora C. WHO indoor air quality guidelines on household fuel combustion: Strategy implications of new evidence on interventions and exposure–risk functions. Atmospheric Environment. 2015;106:451–7. doi: 10.1016/j.atmosenv.2014.08.064

[32] Van Niekerk AV, Reimers A, Laflamme L. Area characteristics and determinants of hospitalised childhood burn injury: a study in the city of Cape Town. Public Health. 2006;120(2):115–24. doi: 10.1016/j.puhe.2005.08.015 16269158

[33] Liao C, Varcoe C, Brown H, Pike I. Beyond individual factors: a critical ethnographic account of urban residential fire risks, experiences, and responses in single-room occupancy (SRO) housing. BMC Public Health. 2024;24(1):2343. doi: 10.1186/s12889-024-19866-z 39198806 PMC11360511

[34] Usman O, Iorember PT, Ozkan O, Alola AA. The asymmetric effect of household and commercial energy efficiency on U.S. energy-related CO2 emissions. Innovation and Green Development. 2025;4(5):100293. doi: 10.1016/j.igd.2025.100293

[35] Juodis A, Reese S. The incidental parameters problem in testing for remaining cross-section correlation. Journal of Business & Economic Statistics. 2022;40(3):1191–203.

[36] Pesaran MH, Xie Y. A bias-corrected CD test for error cross-sectional dependence in panel data models with latent factors. arXiv preprint. 2021. doi: arXiv:2109.00408

[37] Pesaran MH. Testing Weak Cross-Sectional Dependence in Large Panels. Econometric Reviews. 2014;34(6–10):1089–117. doi: 10.1080/07474938.2014.956623

[38] Pesaran MH. General diagnostic tests for cross-sectional dependence in panels. Empir Econ. 2021;60(1):13–50. doi: 10.1007/s00181-020-01875-7

[39] Fan J, Liao Y, Yao J. Power Enhancement in High Dimensional Cross-Sectional Tests. Econometrica. 2015;83(4):1497–541. doi: 10.3982/ECTA12749 26778846 PMC4714420

[40] Pesaran MH. A simple panel unit root test in the presence of cross‐section dependence. J Appl Econ. 2007;22(2):265–312. 10.1002/jae.951

[41] Beck N, Katz JN. What to do (and not to do) with time-series cross-section data. Am Polit Sci Rev. 1995;89(3):634–647. 10.2307/2082979

[42] Williams R. Using the Margins Command to Estimate and Interpret Adjusted Predictions and Marginal Effects. The Stata Journal: Promoting communications on statistics and Stata. 2012;12(2):308–31. doi: 10.1177/1536867x1201200209

[43] StataCorp. Marginsplot-Graph results from margins (profile plots, etc.). College Station, TX: StataCorp. 2013.

[44] Clouston SAP, Link BG. A retrospective on fundamental cause theory: State of the literature, and goals for the future. Annu Rev Sociol. 2021;47(1):131–56. doi: 10.1146/annurev-soc-090320-094912 34949900 PMC8691558

[45] Laflamme L, Hasselberg M, Burrows S. 20 Years of Research on Socioeconomic Inequality and Children’s-Unintentional Injuries Understanding the Cause-Specific Evidence at Hand. Int J Pediatr. 2010;2010:819687. doi: 10.1155/2010/819687 20706660 PMC2913857

[46] Buthelezi SA, Kapwata T, Wernecke B, Webster C, Mathee A, Wright CY. Household Fuel Use for Heating and Cooking and Respiratory Health in a Low-Income, South African Coastal Community. Int J Environ Res Public Health. 2019;16(4):550. doi: 10.3390/ijerph16040550 30769843 PMC6406283

[47] Titi N, van Niekerk A, Ahmed R. Child understandings of the causation of childhood burn injuries: Child activity, parental domestic demands, and impoverished settings. Child Care Health Dev. 2018;44(3):494–500. doi: 10.1111/cch.12484 28718941

