# Supplementary material for: Socio-structural determinants of burn injuries in Africa: The role of social inequality, informal housing, and access to clean cooking energy technology
Source: PLoS One. 2025 Dec 12;20(12):e0336633. doi: 10.1371/journal.pone.0336633 (PMC12700400; doi:10.1371/journal.pone.0336633)
Supplement: S1 Appendix — (DOCX) [file pone.0336633.s001.docx]

S1 Appendix: Variables Description, Measurement and Sources

| **Variable/Acronym** | **Measurement/Description** | **Source** |
| --- | --- | --- |
| Burn Injuries incidence (BUIJinc) | Measured by number or incidence of burn injuries. It is a critical indicator of health outcome. It is a dependent variable. | Institute for Health Metrics and Evaluation; Global Burden of Disease (GBD) https://vizhub.healthdata.org/gbd-results/ |
| Burn Injuries prevalence (BUIJpre) | Measured by number of cases of burn injuries. It is a critical indicator of health outcome. It is a dependent variable. | Institute for Health Metrics and Evaluation; Global Burden of Disease (GBD) https://vizhub.healthdata.org/gbd-results/ |
| Social Inequality (SOCI) | Measured by the Palma Ratio which divides the share of income received by the richest 10% by the share of the poorest 40%. Higher values indicate higher inequality. It is an independent variable. | World Inequality Database (WID), processed by Our World in Data <file:///C:/Users/user/Downloads/palma-ratio-after-tax%20(2).svg> |
| Access to clean energy and technologies for cooking (ACET) | Measured by access to affordable, reliable, sustainable, and modern energy for all (% of population). It is an independent variable. | WDI: [https://databank.worldbank.org/source/world-development-indicators](https://protect.checkpoint.com/v2/___https://databank.worldbank.org/source/world-development-indicators___.YzJlOnVuaXNhbW9iaWxlOmM6bzpkNWE3ZjA0NmIwMjQ2OGNmNzljNjY2ZjgzNDQxNjMzMDo2OmM2ZGY6ZGNkMjI1NjNmNzA1ZGY0OWNmYWY1OTM5NzIzZDg1MzEzNmViMjg1M2YzMjdmOTA1MDE1NWNjNmRmMmNjMTQ5ZDpwOlQ6Tg) |
| Informal/Adequate Housing (IHOS) | Measured by the proportion of urban population living in slums, informal settlements or inadequate housing (%). It is an independent variable. | United Nations – Habitat: [https://data.unhabitat.org/pages/housing-slums-and-informal-settlements](https://protect.checkpoint.com/v2/___https://data.unhabitat.org/pages/housing-slums-and-informal-settlements___.YzJlOnVuaXNhbW9iaWxlOmM6bzpkNWE3ZjA0NmIwMjQ2OGNmNzljNjY2ZjgzNDQxNjMzMDo2OjY5MDg6YzVmNTk3MWU3MWY5MWE4ODFkOGFjMGRmYjAxNTdmODZmZGExM2YwZGRhNjY4NWQzY2RkYjBhZTg0NGM3MDUwNTpwOlQ6Tg) |
| International Energy Trade (INET) | Measured by net energy imports. It is a control variable. | WDI: [https://databank.worldbank.org/source/world-development-indicators](https://protect.checkpoint.com/v2/___https://databank.worldbank.org/source/world-development-indicators___.YzJlOnVuaXNhbW9iaWxlOmM6bzpkNWE3ZjA0NmIwMjQ2OGNmNzljNjY2ZjgzNDQxNjMzMDo2OmM2ZGY6ZGNkMjI1NjNmNzA1ZGY0OWNmYWY1OTM5NzIzZDg1MzEzNmViMjg1M2YzMjdmOTA1MDE1NWNjNmRmMmNjMTQ5ZDpwOlQ6Tg) |
